# In Silico Analysis Identified Putative Pathogenic Missense nsSNPs in Human *SLITRK1* Gene

**DOI:** 10.3390/genes13040672

**Published:** 2022-04-11

**Authors:** Muhammad Zeeshan Ali, Arshad Farid, Safeer Ahmad, Muhammad Muzammal, Mohammed Al Mohaini, Abdulkhaliq J. Alsalman, Maitham A. Al Hawaj, Yousef N. Alhashem, Abdulmonem A. Alsaleh, Eman M. Almusalami, Mahpara Maryam, Muzammil Ahmad Khan

**Affiliations:** 1 Gomal Center of Biochemistry and Biotechnology, Gomal University, Dera Ismail Khan 29111, Pakistan; xeeshan.biotech@yahoo.com (M.Z.A.); arshadfarid@gu.edu.pk (A.F.); safeer9365@gmail.com (S.A.); mustafamuzammal1@yahoo.com (M.M.); 2 Basic Sciences Department, College of Applied Medical Sciences, King Saud Bin Abdulaziz University for Health Sciences, Al Ahsa 31982, Saudi Arabia; mohainim@ksau-hs.edu.sa; 3 King Abdullah International Medical Research Center, Al Ahsa 31982, Saudi Arabia; 4 Department of Clinical Pharmacy, Faculty of Pharmacy, Northern Border University, Rafha 91911, Saudi Arabia; kaliqs@gmail.com; 5 Department of Pharmacy Practice, College of Clinical Pharmacy, King Faisal University, Al Ahsa 31982, Saudi Arabia; hawaj@kfu.edu.sa; 6 Clinical Laboratory Sciences Department, Mohammed Al-Mana College for Medical Sciences, Dammam 34222, Saudi Arabia; yousefa@machs.edu.sa (Y.N.A.); a.alsaleh@machs.edu.sa (A.A.A.); 7 King’s College London, Strand, London WC2R 2LS, UK; eman.al_musalami@kcl.ac.uk; 8 Department of Zoology, Government College No.1, Dera Ismail Khan 29111, Pakistan; mahparamaryam10@gmail.com; 9 Department of Human Genetics, Sidra Medical and Research Centre, Doha 26999, Qatar

**Keywords:** *SLITRK1*, bioinformatical tools, pathogenic, docking

## Abstract

Human DNA contains several variations, which can affect the structure and normal functioning of a protein. These variations could be single nucleotide polymorphisms (SNPs) or insertion-deletions (InDels). SNPs, as opposed to InDels, are more commonly present in DNA and may cause genetic disorders. In the current study, several bioinformatic tools were used to prioritize the pathogenic variants in the *SLITRK1* gene. Out of all of the variants, 16 were commonly predicted to be pathogenic by these tools. All the variants had very low frequency, i.e., <0.0001 in the global population. The secondary structure of all filtered variants was predicted, but no structural change was observed at the site of variation in any variant. Protein stability analysis of these variants was then performed, which determined a decrease in protein stability of 10 of the variants. Amino acid conservation analysis revealed that all the amino acids were highly conserved, indicating their structural and functional importance. Protein 3D structure of wildtype SLITRK1 and all of its variants was predicted using I-TASSER, and the effect of variation on 3D structure of the protein was observed using the Missense3D tool, which presented the probable structural loss in three variants, i.e., Asn529Lys, Leu496Pro and Leu94Phe. The wildtype SLITRK1 protein and these three variants were independently docked with their close interactor protein PTPRD, and remarkable differences were observed in the docking sites of normal and variants, which will ultimately affect the functional activity of the SLITRK1 protein. Previous studies have shown that mutations in *SLITRK1* are involved in Tourette syndrome. The present study may assist a molecular geneticist in interpreting the variant pathogenicity in research as well as diagnostic setup.

## 1. Introduction

**Figure 1:**
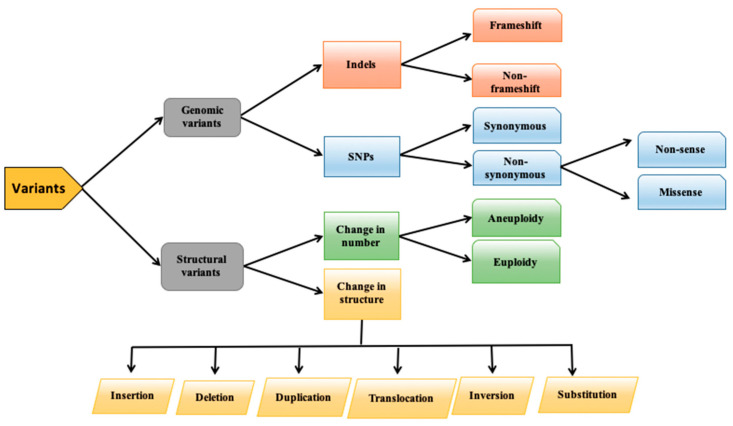
Classification of variants.

Human DNA contains several variations in its sequence including single nucleotide polymorphisms (SNPs) and insertion deletions (InDels). However, SNPs are the most frequently occurring variations in the human genome. These variations in the genome may alter the protein structure and function and can affect the normal character(s) of an organism [1,2,3]. InDels cause substantial genetic variation in the genome of an organism. Many of the InDels occur at the functionally important part of the genome and hence may also play their role in disease onset [4]. Single nucleotide substitution may either cause missense or nonsense effect. The detailed classification of variant is shown in Figure 1. In missense effect, one amino acid is replaced by another amino acid, while the nonsense variants replace the coding codon with a stop codon that eventually leads to the truncation of protein [5]. About 90% of human genome polymorphisms comprises SNPs. Through genome wide prioritization, 0.12% of the variants out of total human genome are predicted to be pathogenic [6]. Several SNPs do not contribute to the causation of disease, but there are certain SNPs that are called missense SNPs or non-synonymous SNPs (nsSNPs) and that are involved in genetic disorders [7]. In about 50% of the total known mutations, nsSNPs are the major contributing factor [8,9].

Most of the disease-causing SNPs are reported at evolutionary-conserved regions of the human genome, which have great importance in the structure and function of proteins. It is very important to identify the pathogenicity of specific SNP for disease prognosis. The identification of SNPs involved in disease is a difficult job, as it requires multiple tests for hundreds to thousands of SNPs in candidate genes. Prioritizing SNPs using bioinformatical tools would be a possible way to overcome this problem. Bioinformatics prediction tools help us to discriminate disease-causing variants from neutral ones.

In the current study, several bioinformatics tools were used to investigate the structural and functional consequences of nsSNPs present in the coding region of the human *SLITRK1* gene. We also predicted the 3D structure of the wildtype SLITRK1 protein and its prioritized predicted pathogenic variants. This is the first in silico study of the human *SLITRK1* gene, which is helpful in predicting pathogenic nsSNPs in the coding region of the *SLITRK1* gene.

## 2. Materials and Methods

### 2.1. Variant Recruitment

**Figure 2:**
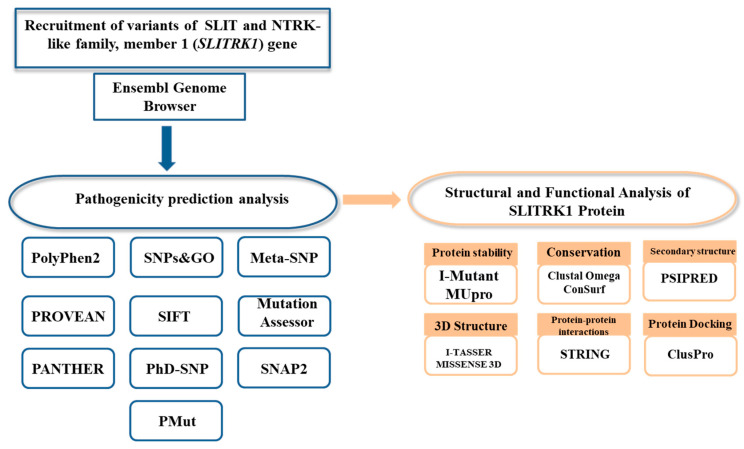
Flowchart of the methodology.

Variants of the *SLITRK1* gene were recruited from the Ensembl genome browser (https://asia.ensembl.org/index.html, accessed on 28 February 2021). Manual variant filtration was performed on an MS Excel supported file enlisting all the variants of the *SLITRK1* gene. Only those variants were selected for further analyses, which were nsSNPs (i.e., missense) and which fell within the coding region of the *SLITRK1* gene. The nsSNPs were analyzed through various bioinformatics tools to find putative pathogenic variants (Figure 2).

### 2.2. Predicting Pathogenicity of Missense nsSNPs

Different online bioinformatics tools were used to predict the pathogenicity of filtered nsSNPs. These online tools were Polyphen2 (Polymorphism Phenotyping v2) (http://genetics.bwh.harvard.edu/pph2/, accessed on 20 March 2021), SNPs&Go (https://snps.biofold.org/snps-and-go/snps-and-go.html, accessed on 15 April 2021), Meta-SNP (https://snps.biofold.org/meta-snp/, accessed on 5 May 2021), PROVEAN (Protein Variation Effect Analyzer) (http://provean.jcvi.org/index.php, accessed on 2 June 2021), SIFT (Sorting Intolerant From Tolerant) (https://sift.bii.a-star.edu.sg, accessed on 27 June 2021), MutationAssessor (http://mutationassessor.org/r3/, accessed on 15 July 2021), PANTHER (Protein ANalysisTHrough Evolutionary Relationships) (http://pantherdb.org, accessed on 2 August 2021), PhD-SNP (Predictor of human Deleterious SNP) (https://snps.biofold.org/phd-snp/phd-snp.html, accessed on 17 August 2021), SNAP2 (https://rostlab.org/services/snap/, accessed on 5 September 2021) and PMut (http://mmb.irbbarcelona.org/PMut/, accessed on 28 September 2021).

### 2.3. Variant Frequency

The frequency of variants that are commonly predicted to be pathogenic by all the bioinformatics pathogenicity predictor tools was checked using dbSNP (https://www.ncbi.nlm.nih.gov/snp/, accessed on 2 October 2021).

### 2.4. Secondary Structure Prediction

The secondary structures of a normal SLITRK1 protein and common predicted pathogenic variants were analyzed through an online tool PSIPRED (http://bioinf.cs.ucl.ac.uk/psipred/, accessed on 4 October 2021).

### 2.5. Protein Stability Analysis

Protein stability of variants was checked using I-Mutant (http://gpcr2.biocomp.unibo.it/cgi/predictors/I-Mutant3.0/I-Mutant3.0.cgi, accessed on 8 October 2021) and MUpro (http://mupro.proteomics.ics.uci.edu, accessed on 9 October 2021). These are web-based tools that predict the stability of a mutated protein.

### 2.6. Conservation Analysis

Conservation analysis was performed using Clustal omega (https://www.ebi.ac.uk/Tools/msa/clustalo/, accessed on 20 October 2021) and ConSurf (http://consurf.tau.ac.il, accessed on 18 January 2022). The amino acid sequence of the SLITRK1 protein of humans and some other species was obtained from the HomoloGene sub-database of NCBI (https://www.ncbi.nlm.nih.gov, accessed on 20 October 2021) and submitted to Clustal Omega (https://www.ebi.ac.uk/Tools/msa/clustalo/, accessed on 20 October 2021). Multiple sequence alignment was performed on Clustal omega to check the conservation of amino acids of the SLITRK1 protein among different species including *Xenopus tropicalis* (western clawed frog), *Gallus* (red jungle fowl), *Bos Taurus* (domestic cow), *Canis lupus familiaris* (domestic dog), *Macaca mulata* (rhesus macaque), *Pan troglodytes* (chimpanzee), *Mus musculus* (house mouse) and *Rattus norvegicus* (brown rat).

The ConSurf web server [10] demonstrates the evolutionary pattern of the amino acids and nucleic acids by predicting the structural and functional areas. The results are predicted based on conservation scores that range from 1 to 9, where 1 indicates variable regions, 5 indicates mild conserved regions and 9 indicates highly conserved regions. However, exposed residues with high scores are considered functional residues, whereas buried residues with high scores are considered structural.

### 2.7. Protein 3D Structure Prediction

I-TASSER (https://zhanglab.ccmb.med.umich.edu/I-TASSER/, accessed on 1 December 2021) tool was used to predict the 3D structure of SLITRK1 protein and its commonly predicted pathogenic variants. Missense3D (http://missense3d.bc.ic.ac.uk/missense3d/, accessed on 10 January 2022) was used to predict structural changes in protein by substitution of an amino acid. UCSF Chimera (candidate version 1.15) was used to visualize the 3D structures of proteins retrieved from I-TASSER. 3D structure of normal and variant proteins was overlapped in UCSF Chimera to observe structural changes.

### 2.8. Protein–Protein Interactions

The interaction of SLITRK1 protein with other proteins was studied using online tool STRING (https://string-db.org, accessed on 20 January 2022), which predicts the top ten proteins that show interactions with the query gene. STRING predicts the interactors of a protein on the basis of gene fusion, co-expression, function and experimental data. It shows combined scores for each interacting protein, ranging from 0 to 1, where 0 shows the lowest interaction and 1 indicates the highest interaction [11].

### 2.9. Protein-Protein Docking

The online tool Cluspro was used for docking of the normal and mutant SLITRK1 proteins with its close functional interactor [12].

## 3. Results

### 3.1. Variant Recruitment

The total identified variants in SLITRK1 include 07 indels, 321 5′UTR and 624 3′UTR variants, and 5 nonsense variants. A total of 2255 variants were recruited from the Ensembl genome browser, which consisted of synonymous, non-synonymous, intronic, 3′UTR and 5′UTR variants. Out of the nonsynonymous variants, 7 were indels and 447 were SNPs. Of these nsSNPs, 442 variants were missense, i.e., causing change in amino acid, and 5 variants resulted in the formation of a stop codon, causing truncation of the protein. Only missense nsSNPs were selected for further bioinformatical analysis.

### 3.2. Pathogenicity Prediction of Variants

All the missense nsSNPs were subjected to ten bioinformatical tools to predict their biological pathogenicity. The proportion of pathogenic variants predicted by different software include 146 by Polyphen2, 101 by SNPs&Go, 102 by Meta-SNP, 103 by PROVEAN, 177 by SIFT, 104 by MutationAssessor, 395 by PANTHER, 141 by PhD-SNP, 160 by SNAP2 and 387 by PMut. Among all the variants, 16 were commonly predicted to be pathogenic by all the ten bioinformatical tools (Table 1). These 16 variants were then finally selected for further analysis due to their high susceptibility for being pathogenic.

### 3.3. Variant Frequency

dbSNP (https://www.ncbi.nlm.nih.gov/snp/, accessed on 2 October 2021) was used to check the frequency of commonly predicted pathogenic variants. The frequency of all the variants was very low (<0.0001) in the global population. This supported the findings produced by pathogenicity prediction tools.

### 3.4. Secondary Structure Prediction

The secondary structure predictions indicated that all the selected variants lie in the coil of the SLITRK1 protein and only one lies in the helix. There was no structural change observed at the point of change. However, some upstream and downstream changes were observed.

### 3.5. Protein Stability Analysis

I-Mutant and MUpro tools were used to check the stability of the SLITRK1 protein for selected amino acid substitutions. Among 16 commonly predicted pathogenic variants, 10 of the variants were commonly predicted to show decreases in the stability of the protein, which suggestively may cause greater loss to the SLITRK1 protein (Table 2).

### 3.6. Amino Acid Conservation

#### 3.6.1. Clustal Omega

**Figure 3:**
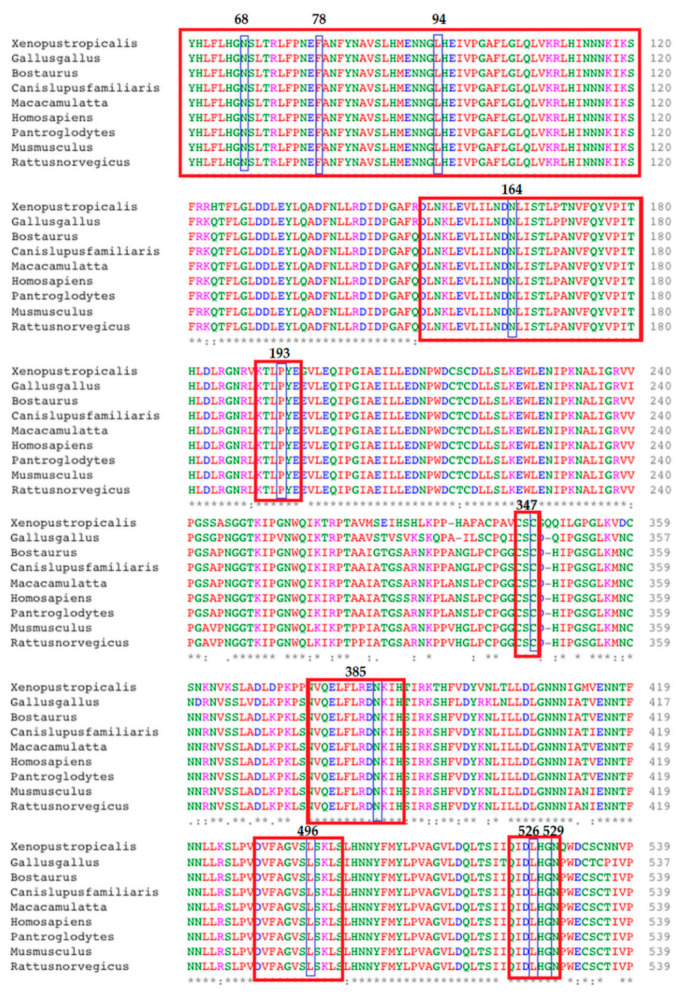
Amino acid conservation analysis using Clustal Omega. Note: Conserved regions harboring the SNP of interest are highlighted by the red box.

The Clustal Omega tool was used for multiple sequence alignment of the SLITRK1 protein of humans with other species. The results revealed that all the amino acids at the point of variations were highly conserved in all other species, indicating the evolutionary and functional importance of selected amino acids (Figure 3).

#### 3.6.2. ConSurf

**Figure 4:**
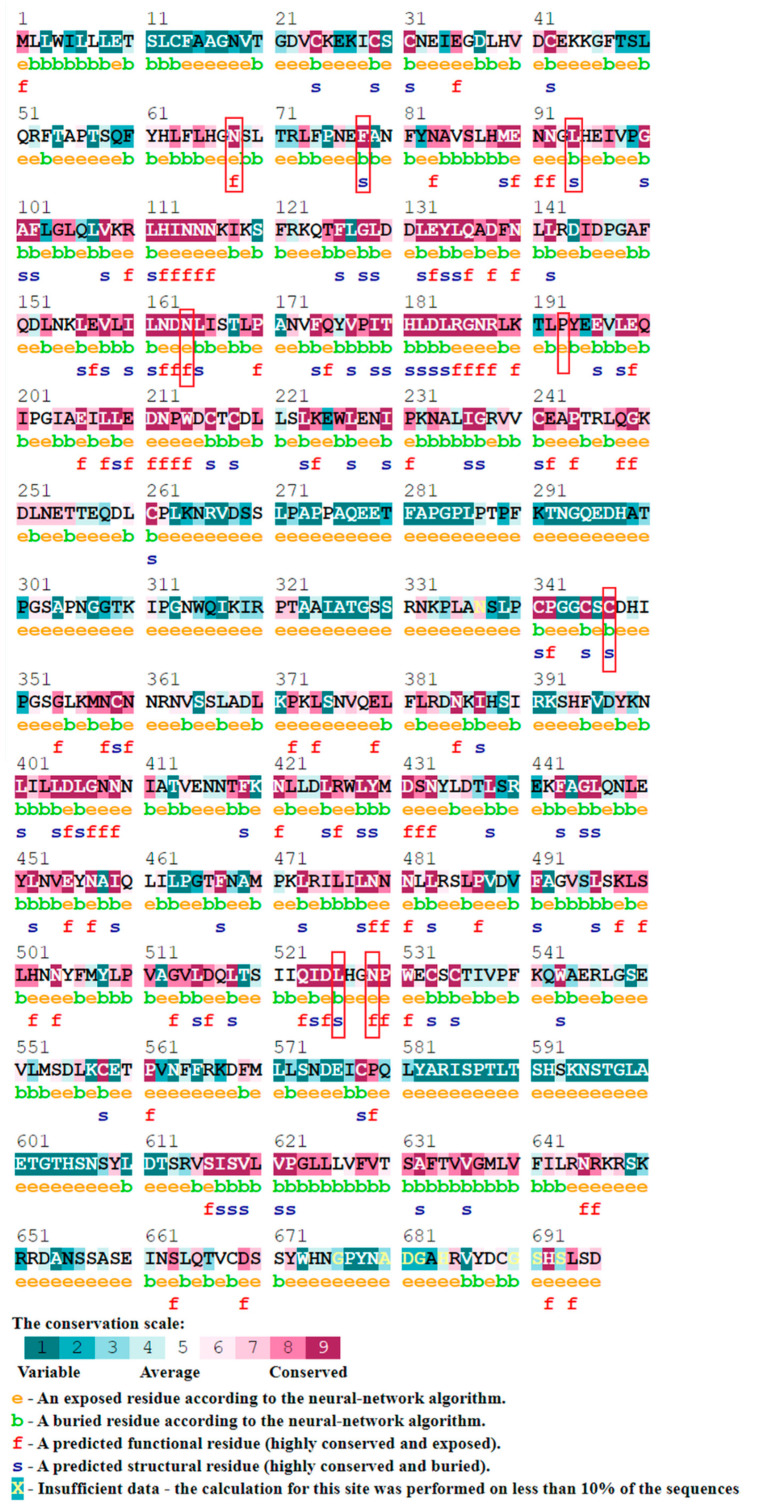
Evolutionary conservation analysis using ConSurf.

The ConSurf tool was used to identify the evolutionary conservation of amino acids of the SLITRK1 protein. ConSurf predicts which amino acids play structural or functional roles based on conservation and solvent accessibility. Residues are predicted as being functional when they are highly conserved and exposed and as structural when they are highly conserved and buried. The results indicate that all 10 nsSNPs that are predicted to be damaging are highly conserved i.e., nine variants having a conservation score of 9 and 1 showing a conservation score of 8. Of the above 10 amino acids, half were buried and predicted as structural residues while the rest were exposed and predicted as functional residues (Figure 4).

### 3.7. 3D Structure Predictions

**Figure 5:**
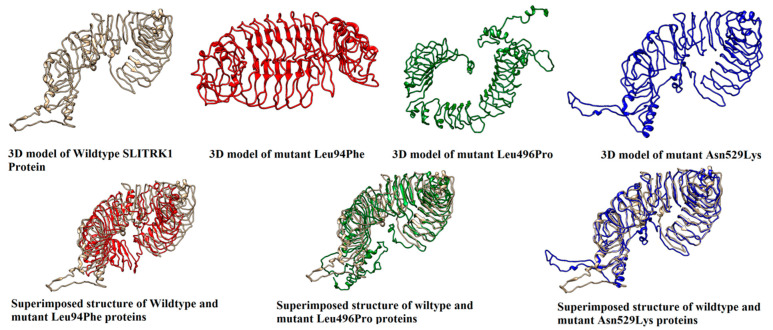
Three-dimensional structure of the SLITRK1 wild type protein, its variants, and the superimposed structures of the wild type and variants.

Three-dimensional models of all SLITRKI variants were designed and superimposed with the 3D structure of wild SLITRK1 (Figure 5). The Missense3D tool was used to detect the structural changes that were caused due to substitution of the amino acids. The Missense3D tool predicted structural damage in variants I, III and VIII, while no structural damage was detected in the other variants. Hence, we selected variants I, III and VIII for further analysis. By manual comparison of all the structures, different changes in the folding pattern of protein were absorbed. The highest similarity index of 71.12% was shown in the 3D model of the mutant Asn529Lys with the wildtype 3D model of the SLITRK1 protein, while the lowest similarity index was shown in the 3D model of mutant Leu94Phe with the wildtype 3D model of the SLITRK1 protein. The similarity index of the 3D model of mutant Leu496Pro with the wildtype SLITRK1 protein was 31.32%.

### 3.8. Protein–Protein Interactions

**Figure 6:**
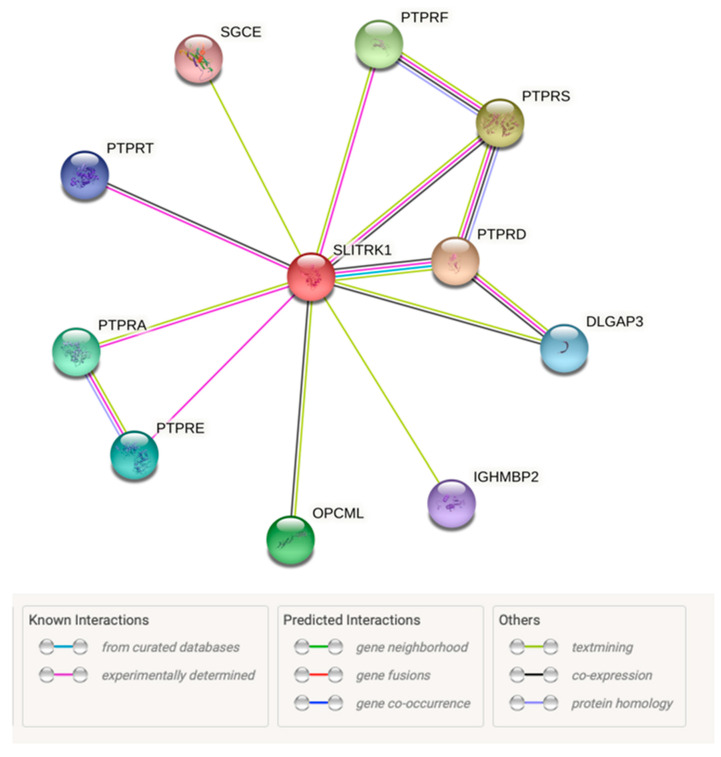
Prediction of protein–protein interaction using STRING.

The String tool was used to predict the close interactor protein of SLITRK1. The results showed that SLITRK1 has close interactions with the PTPRD, PTPRS, PTPRF, OPCML, PTPRA, PTPRE, DLGAP3, PTPRT, IGHMBP2 and SGCE proteins. However, the SLITRK1 protein determined PTPRD as being the closest functional interactor (Figure 6).

### 3.9. Protein–Protein Docking

The wildtype SLITRK1 protein and all its variants were docked with the close interactor PTPRD protein and notable differences were observed in the interacting sites of wildtype SLITRK1 and its variants with PTPRD. The results showed that wildtype SLITRK1 has interactions with PTPRD at eight amino acid residues, i.e., Ala680, Lys647, Phe641, Arg584, Thr604, Tyr582, Glu550 and Ser616. These interactions are through 8 interactive forces including 7 H-bonds and 1 unfavorable bond.

**Figure 7:**
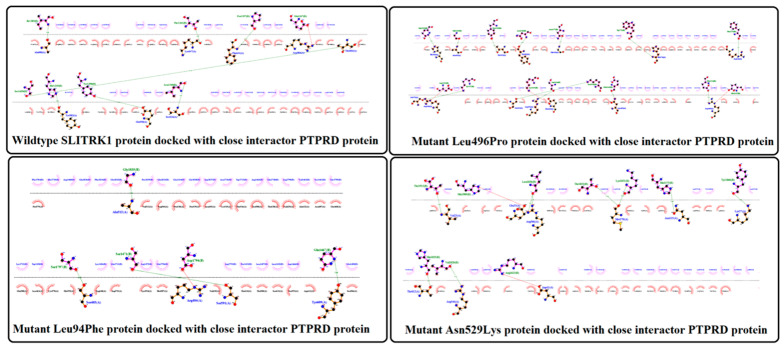
Protein–Protein docking of the SLITRK1 wild type and variants with their close interactor PTPRD.

Variant Leu94Phe showed the lowest interaction with PTPRD, i.e., interacting at five different residues via four H-bonds and one unfavorable bond, while variant Leu496Pro showed the highest interaction with PTPRD, interacting at 14 different residues via 15 bonds, including 12 H-bonds and 3 unfavorable bonds. The interactions of SLITRK1 (normal and all variants) with close interactor PTPRD are diagrammatically shown in Figure 7.

## 4. Discussion

SNPs, also known as single nucleotide variants (SNVs), are the most commonly found variants in the human genome. According to an estimate, the human genome contains at least 11 million SNPs (1 per 300 bp on average) [13]. SNPs are found in protein coding as well as in non-protein coding regions [14,15]. Research has shown that variations in non-coding elements may also be the cause of several genetic conditions [16]. Numerous evidence has shown that there are some variants that are found in functional non-coding regions including chromatin marks, DNase hypersensitivity and enhancer elements [17,18]. The role of variations in non-coding regions including intergenic sequence, non-coding RNAs and non-coding elements in protein coding gene, is challenging to determine and needs to be better understood. The non-coding variants involved in causation of different disorders may be found in enhancer regions, promoter sites, or 5′UTR or 3′UTR of the gene [19]. Another important role of the non-coding variant is in the regulation of gene expression, which is a challenging task when identifying the effect of variation in molecular mechanism of gene regulation [20]. Studies have also demonstrated that the role of variations in non-coding regions are associated with the timing of DNA replication [21]. Variations in the non-coding genome are associated with various diseases, but to fully understand their functional effects, much research is still required.

In the human genome, around 24,000 to 60,000 coding SNPs are estimated [22,23]. nsSNPs are more significant because they have the potential to affect the structure and function of expressed proteins and are, therefore, likely to represent modifiers of inherited susceptibility to disease [24]. nsSNPs alter cellular functions in many ways. Indeed, nsSNPs often influence normal protein function through a combination of effects on protein stability, protein–protein interactions and many other features [25]. Numerous studies in the past have shown that nsSNPs are responsible for about 50% of mutations that are involved in various genetic disorders [9]. This information confirms that nsSNPs, especially missense SNPs, are associated with various human diseases. Recent studies on the nsSNPs using computational approaches reveal the potential impact of mutation on understanding the molecular mechanisms of various diseases [26,27,28].

SLITRK1 is a member of the SLITRK family and, similar to other members of the SLITRK family, is an integral membrane protein with the domain ‘2 N-terminal leucine rich repeat (LRR) [29]. SLITRK1 has the LRP1 domain, through which they interact with LAR receptor protein tyrosine phosphatases (PTPs) and control synapse formation [30,31,32].

The *SLITRK1* gene was mapped to a region of chromosome 13q31 [29,30,31,32,33]. The *SLITRK1* gene is highly expressed in adult and fetal brains; moderately expressed in lungs and pancreas; and has very low expressions in the ovaries, kidneys, heart and liver [33]. Mouse *Slitrk1* cDNA was cloned by Aruga and Mikoshiba (2003), who discovered that the protein contains a signal peptide at *N*-terminus, which was followed by LRR domains and a transmembrane domain at the *C*-terminus [29]. Aruga and Mikoshiba (2003) performed a Northern blot analysis of various mouse tissues and found a very high expression of Slitrk1 only in the brain [29]. Until now, 14 different types of mutations have been identified in the *SLITRK1* gene (HGMD). Mutations in the *SLITRK1* gene have been reported to be involved in Tourette Syndrome (TS; OMIM No# 137580) [34]. TS is a neuropsychiatric disorder with an estimated onset in early childhood. It is characterized by vocal and motor tics. It prevails in 1 out of every 100 individuals worldwide. TS patients often have obsessive compulsive disorder (OCD) as well as attention deficit hyperactivity disorder (ADHD) along with some disorders of mood, sleep, depression and anxiety [35,36]. Many genes (such as *NTN4*, *SLC6A4*, *IMMP2L*, *CNTNAP2*, *NLGN4*, *HDC* and *SLITRK1*) and some chromosomal loci have been known to date to be involved in TS [37]. The dysfunction of serotonin and dopamine neurotransmitters and defects of cortico-striatal-thalamic-cortical pathways are considered to have an association with TS. Despite extensive research in genetics, the pathogenetic mechanism of TS is still largely lacking and the number of variants likely to cause TS is extremely small [38,39].

Here, in the current study, we performed a bioinformatical approach to predict the probably harmful nsSNPs and their possible consequences on the structure and function of SLITRK1 proteins. The total identified variants in *SLITRK1* include 07 indels, 321 5′UTR and 624 3′UTR variants, and 05 nonsense variants. The analysis initially identified 442 missense variants out of 2255 total variants. Different pathogenicity prediction tools commonly predicted 16 variants to be presumably harmful for protein structure and function. The frequency of all 16 variants was very low. The secondary structure of these 16 variants did not show any change at the site of variation. A protein stability analysis is necessary to assess the structural and functional activity of a protein [40]. Protein stability governs the conformational structure of the protein and thus determines the function. Any alteration in protein stability may cause miss-folding, degradation or aberrant accumulation of proteins [41]. Out of 16 variants, 10 variants showed decreases in the protein stability based on the I-Mutant and MuPro tools. The conservation of amino acids at the points of substitutions was checked in nine different species including Homo sapiens using the Clustal Omega tool. The results showed that all the amino acids at the site of variations were highly conserved, indicating their structural and functional importance. The Missense 3D tool was used to check the possible loss in the 3D structure of the SLITRK1 protein, which is caused by a substitution of amino acids. The results showed that variants I, III and VIII affect the structural confirmation of SLITRK1 protein. Hence, variants I, III and VIII were selected for further in silico analysis. The wildtype SLITRK1 protein and its three variants i.e., Leu94Phe, Leu496Pro and Asn529Lys were docked with the close interactor protein PTPRD. Molecular docking analysis revealed that the aforementioned variants can possibly affect the functional activity of the SLITRK1 protein.

The limitation of current study is that the analysis was conducted without considering the disease model because a single gene may independently cause different diseases with different segregation patterns. Further to this, the alleles (obtained from genome browser for current analysis) exists in real form; however, until now, it was not associated with any disease onset due to several reasons, such as the allele being associated with some recessive condition where the presence of homozygous genotype would be necessary for disease onset. Nevertheless, the allele floats in the population, which may coincidently or due to extensive consanguinity, unite in a single individual (i.e., homozygous genotype) and cause the disorder. Therefore, in this study, we tried to focus on predictions of the detrimental effect of the allele regardless of disease inheritance pattern or disorder type. Hence, it is speculated that, if this allele segregates in an individual or family, it may have a negative impact. Moreover, the study also did not include the non-coding variants in spite of their significant role in the spatio-temporal gene expression pattern.

Conclusively, the present bioinformatic study would assist a molecular geneticist in interpreting the variant pathogenicity in research as well as diagnostic setup.

## Figures and Tables

**Table 1:** List of variants commonly predicted to be pathogenic by all tools.

S.No	Chr:bp	Alleles	AA	AA Coord	Polyphen2		SNPs&Go		MetaSNP		Provean		SIFT		Mutation Assessor		Panther		PHD SNP		SNAP2		PMut	
					Pred	Prob	Pred	Prob	Pred	Score	Pred	Score	Pred	Score	F I	FI Score	Pred	Preservation Time	Pred	Score	Pred	Score	Pred	Score
1	13:83879772	G/A	Pro/Leu	579	ProD	0.997	D	0.736	D	0.657	D	−8.14	D	0	M	2.995	ProD	750	D	8	E	5	D	0.6827
2	13:83879921	G/T	Asn/Lys	529	ProD	1	D	0.853	D	0.81	D	−5.77	D	0	M	3.065	ProD	456	D	8	E	82	D	0.7961
3	13:83879932	G/A	Leu/Phe	526	ProD	0.997	D	0.673	D	0.603	D	−3.85	D	0	M	2.74	ProD	750	D	7	E	71	D	0.7852
4	13:83879997	T/A	Asn/Ile	504	ProD	0.999	D	0.907	D	0.825	D	−8.14	D	0	H	4.565	ProD	750	D	8	E	85	D	0.8016
5	13:83880021	A/G	Leu/Pro	496	ProD	1	D	0.834	D	0.786	D	−6.15	D	0	H	4.75	ProD	456	D	6	E	90	D	0.8058
6	13:83880024	G/C	Ser/Trp	495	ProD	0.985	D	0.616	D	0.72	D	−4.2	D	0.01	H	4.165	ProD	456	D	1	E	65	D	0.7634
7	13:83880354	T/C	Asn/Ser	385	ProD	0.999	D	0.812	D	0.721	D	−4.37	D	0	M	2.995	PosD	361	D	8	E	72	D	0.5334
8	13:83880432	C/T	Cys/Tyr	359	ProD	0.999	D	0.922	D	0.816	D	−9.47	D	0	M	2.62	PosD	361	D	4	E	82	D	0.8359
9	13:83880435	T/A	Asn/Ile	358	PosD	0.775	D	0.636	D	0.761	D	−6.24	D	0	M	2.3	PosD	361	D	3	E	58	D	0.7522
10	13:83880469	A/C	Cys/Gly	347	ProD	1	D	0.757	D	0.733	D	−9.53	D	0	M	2.62	ProD	750	D	6	E	86	D	0.7549
11	13:83880930	G/A	Pro/Leu	193	ProD	0.949	D	0.767	D	0.676	D	−6.7	D	0	M	2.93	ProD	750	D	8	E	17	D	0.6558
12	13:83881017	T/C	Asn/Ser	164	ProD	1	D	0.833	D	0.766	D	−4.75	D	0	M	3.375	ProD	750	D	8	E	66	D	0.7106
13	13:83881162	T/A	Asn/Tyr	116	ProD	1	D	0.907	D	0.865	D	−7.59	D	0	H	4.72	ProD	750	D	7	E	83	D	0.7989
14	13:83881226	C/A	Leu/Phe	94	ProD	0.98	D	0.744	D	0.532	D	−2.96	D	0.01	M	3.18	ProD	456	D	7	E	46	D	0.8303
15	13:83881276	A/G	Phe/Leu	78	PosD	0.831	D	0.741	D	0.511	D	−4.8	D	0.02	M	2.035	ProD	750	D	8	E	52	D	0.5225
16	13:83881305	T/C	Asn/Ser	68	ProD	0.985	D	0.753	D	0.69	D	−4.72	D	0	H	3.555	ProD	750	D	1	E	64	D	0.8179

Key: Chr: Chromosome, bp: Base pair, AA: Amino Acid, AA coord: Amino Acid Coordinate, Pred: Prediction, Prob: Probability, FI: Functional Impact, Pro D: Probably damaging, Pos D: Possibly damaging, D: Damaging, M: Medium, H: High, E: Effect.

**Table 2:** List of variants showing decreases in the protein stability.

Variant No.	rs ID	AA	AA Coord	I-Mutant		MuPro	
				Stability	RI	Stability	Score
I	rs1048143268	Asn/Lys	529	Decrease	4	Decrease	−0.86
II	rs1219903976	Leu/Phe	526	Decrease	8	Decrease	−0.99
III	rs1226852299	Leu/Pro	496	Decrease	6	Decrease	−0.992
IV	rs1472728808	Asn/Ser	385	Decrease	5	Decrease	−0.971
V	rs1277399796	Cys/Gly	347	Decrease	7	Decrease	−0.994
VI	rs1429907885	Pro/Leu	193	Decrease	6	Decrease	−0.778
VII	rs774612607	Asn/Ser	164	Decrease	5	Decrease	−0.999
VIII	rs1410244448	Leu/Phe	94	Decrease	8	Decrease	−0.999
IX	rs954218528	Phe/Leu	78	Decrease	7	Decrease	−0.661
X	rs1228122404	Asn/Ser	68	Decrease	2	Decrease	−0.751

## Data Availability

The computational data are stored in the password-protected personal computers of M.M. and M.A.K., which is available to the editors upon request.
